# Viral Myocarditis: Classification, Diagnosis, and Clinical Implications

**DOI:** 10.3389/fcvm.2022.908663

**Published:** 2022-06-20

**Authors:** Fabiola B. Sozzi, Elisa Gherbesi, Andrea Faggiano, Eleonora Gnan, Alessio Maruccio, Marco Schiavone, Laura Iacuzio, Stefano Carugo

**Affiliations:** ^1^Cardiology Unit, Internal Medicine Department, Fondazione Ospedale Maggiore Policlinico IRCCS Cà Granda, University of Milan, Milan, Italy; ^2^Cardiology Unit, Luigi Sacco University Hospital, Milan, Italy; ^3^CCM Centre Cardiothoracique, Monaco, Monaco

**Keywords:** viral myocarditis, cardiac magnetic resonance, arrhythmias, ICD (implantable cardioverter-defibrillator), sudden cardiac arrest (SCA)

## Abstract

Myocarditis is an inflammatory disease of the myocardium with focal or diffuse involvement. Viral infections are the most common cause of myocarditis, especially in Western countries. A recent viral illness with gastroenteric or upper respiratory symptoms often precedes myocarditis. The absence of specific pathognomonic features in conjunction with the wide spectrum of clinical manifestations that range from subclinical cases to sudden cardiac death (SCD) makes myocarditis diagnosis particularly challenging. Moreover, myocarditis might represent a cause of initially unexplained dilated cardiomyopathy (DCM) and heart failure (HF), especially among children and young adults. Cardiac magnetic resonance imaging (CMR) is crucial for myocarditis diagnosis, because of its ability to detect interstitial edema during acute inflammation. Assessment of subepicardial or mid-myocardial fibrosis by late gadolinium enhancement (LGE) is typical for myocarditis. Cardiac arrhythmias are frequent events that may arise especially in more severe myocarditis cases. The most common form of arrhythmia is atrial fibrillation, followed by ventricular tachycardia. Documented arrhythmias have been reported more commonly with HIV myocarditis than other more common infections such as Adenovirus, Parvovirus B19, human Herpes virus 6, and Enterovirus. The mechanisms of arrhythmogenesis in myocardial inflammation are not fully understood; in the acute phase, the spectrum of arrhythmogenesis ranges from a direct effect on cardiomyocytes that leads to electrical instability and ion channel impairment to ischemia from coronary macro- or microvascular disease. In chronic myocarditis, instead, myocardial replacement with fibrosis promotes scar-mediated re-entrant ventricular arrhythmias. Observational data suggested the important role of CMR, with LGE being the strongest independent predictor of SCD, cardiac, and all-cause mortality. In acute myocarditis, the most common localization of subepicardial LGE dwells in the lateral wall. Patients with myocarditis that develop HF and arrhythmias usually show a larger LGE distribution involving several myocardial segments. Moreover, a mid-layer LGE in the interventricular septum is more frequent in acute myocarditis than in acute coronary syndromes cases. The risk of SCD in patients with wide areas of LGE is significant, and a shared decision-making approach is warranted. Nevertheless, there is no formal consensus about the extension of LGE to justify implantable cardioverter defibrillator (ICD) implantation in primary prevention.

## Introduction

Myocarditis is a relatively common but potentially life-threatening inflammatory disease of the myocardium, as defined by established histological, immunological, and immunohistochemical criteria. It affects millions of people worldwide, especially children and male young adults, and represents a relevant cause of sudden cardiac death (SCD), initially unexplained dilated cardiomyopathy (DCM), and heart failure (HF) in these populations (1, 2).

Endomyocardial biopsy (EMB) represents the diagnostic gold standard, but it is underutilized in clinical practice; thus, diagnosis is often drawn from a combination of compatible clinical presentation, non-invasive biomarkers, and imaging features (2).

Cardiac magnetic resonance (CMR) imaging has emerged as the non-invasive reference technique for the diagnosis and follow-up of patients with myocarditis. The accuracy and reproducibility of cardiac structure evaluation, the unique ability of non-invasive tissue characterization, and the lack of ionizing radiation make CMR very attractive as a potential “all-in-one technique”: It provides valuable data to confirm or establish the diagnosis of myocarditis, screen subclinical cases, stratify patients for risk according to established independent prognostic factors (e.g., left ventricular ejection fraction, end-systolic-volume, and extent of myocardial edema), predict the prognosis, and monitor the response to therapy during follow-up.

In this review, we discuss the current state of classification, clinical impact, and treatment of myocarditis. The role of non-invasive imaging in comprehensive evaluation of patients with myocarditis is analyzed, highlighting the pivotal function of CMR in guiding diagnosis and assessing prognosis. Moreover, we aim at providing a state-of-the-art overview of the role of arrythmias in the setting of myocarditis in terms of their prevalence, possible pathogenic mechanisms, short- and long-term prognostic impacts, and treatment.

## Classification

Myocarditis represents a polymorphic and complex entity, as reflected by the multitude of ways in which it can be classified. For instance, it is possible to recognize lymphocytic, eosinophilic, and giant cell or granulomatous myocarditis in relation to the predominant infiltrating cell type at EMB, while according to the underlying etiopathogenic mechanism, the disease can be differentiated into infectious and non-infectious forms. The latter are overall less frequent and include toxic myocarditis (caused by drugs, toxins, or physical agents) and immune-mediated myocarditis, which in turn can result from exposure to allergens, alloantigens, or autoantigens (as in giant cell myocarditis or myocarditis associated with systemic autoimmune diseases) (2).

Infectious myocarditis, on the other hand, can be caused by several pathogens whose relative frequency varies regionally. In resource-limited areas, the disease is often associated with specific conditions such as rheumatic disease, Chagas disease, HIV, and helminthic or bacterial infections (3). Overall, in Western countries, viruses are presumably the most common cause of myocarditis.

Table 1 lists some most relevant implicated viruses. Notably, approximately 27% of patients may present with multiple myocardial viral infections (4).

**TABLE 1:** List of the most common viruses causing myocarditis.

	• Primarycardiactrophism • Most common pathogens in the 1980s–1990s
Human Herpesvirus 6 (HHV6), PARVOVIRUS B-19 (B19V)	• Vascular or lymphatictrophism • Possible lifelong myocardial persistence • Most common pathogens in the past two decades
HIV, HCV, Influenza A and B viruses	• Indirect myocardial damage *via* activation of the immune system
SARS-CoV-2 virus	• Emerging pathogen • Unclear mechanism of myocardial damage • Also vaccine-related

## Pathogenesis

A three-phase model for the pathogenesis of myocarditis (viral myocarditis in particular) has been conceptualized on the basis of clinical observations and animal model data (2).

### Acute Infectious Phase

The first phase of the disease lasts from 1 to 7 days and consists of acute cardiac cell damage and death, subsequent exposure of host proteins, and activation of innate immune response.

The mechanisms of acute myocardial injury may be direct or indirect and vary depending on the causative agent involved. Adenoviruses and enteroviruses, for instance, are cytolytic viruses that enter myocytes through the same transmembrane receptor [the coxsackievirus and adenovirus receptor (CAR)] and cause severe cytopathic effects through various mechanisms, including viral protease 2A-mediated cleavage of the host protein dystrophin (5).

Parvovirus B19 (B19V), on the other hand, is able to infect endothelial cells and trigger the release of pro-inflammatory cytokines through the viral protein NS1. The actual role of B19V as a causative agent of myocarditis is, however, still debated (6).

Finally other viruses, including influenza viruses, can indirectly cause myocarditis by activation of self-reactive T cell response, owing to molecular mimicry between viral and cardiac antigens (3).

The innate immune system plays a critical role in eradicating a viral infection, but excessive or persistent response contributes to significant tissue damage. Different cell subsets have been variably implicated in the first phase of the disease, including neutrophils, natural killer cells, pro-inflammatory and anti-inflammatory macrophages, and dendritic cells.

### Subacute Immune Phase

In the second phase, which lasts from 1 to 4 weeks, disease progression is driven by an adaptive, primarily T cell-mediated, immune response. Studies have underscored the complex balance between the possible beneficial and detrimental effects of both CD4 + and CD8 cells, while limited data are available on the role of B cells in the pathogenesis of viral myocarditis (3).

### Recovery or Chronic Myopathic Phase

Complete elimination of pathogens from the myocardium usually restores cardiac function without leaving residual injury. In genetically susceptible individuals, however, persistent myocardial infection and/or breakdown of T-cell tolerance to cardiac antigens may lead to chronic inflammation, adverse remodeling, development of DCM, and, ultimately, end-stage HF.

Th17 cells are believed to be majorly implicated in progression to inflammatory cardiomyopathy, as demonstrated by an animal study in which IL-17-deficient mice experienced almost the same degree of acute inflammation as wild-type controls but developed less cardiac fibrosis (7). Regulatory T (Treg) cells, on the other hand, are necessary for induction and maintenance of peripheral tolerance and were found to be reduced in patients with myocarditis or inflammatory cardiomyopathy (8).

### Knowledge Gaps and Future Directions

The mechanisms underlying the pathogenesis of myocarditis and its variable progression to chronic disease are far from being fully understood. A favorable genetic background may predispose to serious and persistent disease, as shown in a report on 36 patients with biopsy-proven active lymphocytic myocarditis, of which 31% were found to be carriers of pathogenic or likely pathogenic variants of cardiomyopathy-related genes such as Titin, Desmoplakin, and Filamin C (9).

Owing to their importance in the subacute and chronic phases, a better understanding of the role of various CD4 + T-cell populations and of factors modulating T-cell-related autoimmunity is also needed, as it could provide a basis for promising therapeutic strategies.

In this regard, microRNAs (miRNAs) have emerged as important epigenetic immune response regulators. In studies on EMB samples, patients with myocarditis expressed different miRNA profiles with respect to control subjects, and so did patients with persistent CVB3 infection and progressive cardiac dysfunction compared to patients who experienced spontaneous viral clearance and recovery from myocarditis (10).

**FIGURE 1:**
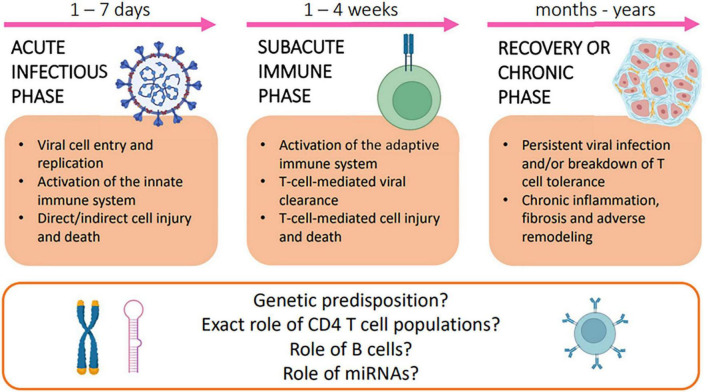
Three-phase model for the pathogenesis of myocarditis.

Figure 1 summarizes the three-phase model for the pathogenesis of myocarditis.

## COVID-19 and Myocarditis

Numerous case reports have described clinical suspicion of myocarditis in patients with coronavirus disease 2019 (COVID-19), including fulminant forms (11). If cardiac injury has been reported in 19–28% of patients diagnosed with COVID-19 (12), associated with worse outcomes, the true epidemiology of COVID-19 myocarditis is difficult to establish. Indeed, cardiac troponin elevation (defined as myocardial injury) may lose its prognostic value in some settings potentially being a bystander, especially in patients with chronic coronary syndromes (13). In a retrospective cohort study (14) using electronic medical records from a global health research network, of 718,365 patients with COVID-19, 5% developed new-onset myocarditis and in this group, 6-month all-cause mortality was 3.9%. From the data of a large hospital-based administrative database of healthcare that encounters from > 900 hospitals in the United States emerged that, during March 2020–January 2021, patients with COVID-19 had nearly 16 times the risk for myocarditis compared with patients who did not have COVID-19; moreover, myocarditis inpatient encounters were 42.3% higher in 2020 (4,560) than in 2019 (3,205). Peaks in myocarditis inpatient encounters during April– May 2020 and November 2020–January 2021 generally aligned with peaks in COVID-19 inpatient encounters (15).

In a large multinational database of patients with COVID-19, as to the propensity-matched cohorts (patients with COVID-19 with and without myocarditis during hospital stay), the all-cause mortality was 13.4 versus 4.2%, respectively, at 30 days (16).

The pathogenesis of acute myocardial damage in COVID-19 is not well-established: direct role of angiotensin-converting enzyme 2 receptors (17) (known enter site of the virus in various cells including macrophages and expressed in myocytes as well) and hyperimmune response (18) are the two main theories.

In a recent systematic review (19) including case reports with laboratory-confirmed COVID-19 and a clinical and/or histological diagnosis of myocarditis by ESC criteria, 38 cases were included: if histological data were available in 12 cases (8 EMB and 4 autopsies), CMR was the main imaging modality to confirm a diagnosis of myocarditis (25 patients). EMB evidence of SARS-CoV-2 cardiotropism has been demonstrated, with virus genome detection in 5 of 104 EMBs of patients with suspected myocarditis or unexplained HF (20).

Besides acute disease, a matter of concern is cardiovascular sequelae even without clinical manifestation of acute myocarditis: in a cohort of German patients (21) who recovered from COVID-19 infection, CMR revealed cardiac involvement in 78% and ongoing myocardial inflammation in 60% (identified with raised myocardial native T1 or T2 mapping or myocardial late gadolinium enhancement) independent of pre-existing conditions, severity, and overall course of the acute illness and time from the original diagnosis. Long-term follow-up for the prognosis of these alterations is needed.

Although rare, acute myocarditis can also occur after vaccination against COVID-19, especially with vaccines based on mRNA technology, with the mechanism not clearly defined but likely due to immune response (22).

The three main mechanisms by which COVID-19 mRNA vaccines might induce hyperimmunity are mRNA immune reactivity, antibodies to SARS-CoV-2 spike glycoproteins cross-reacting with myocardial proteins, and hormonal differences. The immune system might detect the mRNA in the vaccine as an antigen, resulting in activation of proinflammatory cascades and immunological pathways in the heart; molecular mimicry between the spike protein of SARS-CoV-2 and cardiac self-antigens is another possible mechanism; finally, given the increased incidence among male patients, differences in hormone signaling might be involved, as testosterone can inhibit anti-inflammatory immune cells and promote a more aggressive immune response by Th1-lymphocytes (23, 24).

The incidence of myocarditis associated with COVID-19 mRNA vaccination seems to be low, and it has been estimated as.3–5 cases per 100,000 vaccinated people in case-series studies from the United States (25) and Israel (26), with the highest incidence of myocarditis occurring within the first week after the second dose mostly in young men with mild and self-limited illness. However, COVID-19 myocarditis is estimated to be 100 times higher (1,000–1,400 per 100,000 people) than that of COVID-19 vaccine-related one and, in contrast to the overall mild presentation and good outcome of vaccine-associated myocarditis, COVID-19 is associated with higher risk of complications (27).

## Clinical Presentation

Myocarditis more typically affects young adult males and may show a wide spectrum of presenting symptoms and signs, ranging from subclinical or uncomplicated diseases to complicated forms and even SCD. This variability reflects the wide range of possible histologic findings, etiologies, and stages of the disease at presentation.

Chest pain is the most frequent reported symptom (up to 95% of cases) according to large registries, followed by dyspnea (up to 49% of cases). Other typical but non-specific symptoms include fatigue, palpitations, and syncope. A prodrome of fever, flu-like, or gastrointestinal symptoms is reported in 18–80% of patients (28).

According to main scientific societies (2, 29), myocarditis is defined acute when it comes to medical attention within 3 months from symptom onset, even if a distinction between acute (<1 month) and subacute (1–3 months) forms has been proposed by others (28).

Acute forms of myocarditis generally show one of three main clinical profiles, as described by the 2013 European Society of Cardiology position statement (2):

- Acute coronary syndrome-like presentation, with chest pain, ST/T wave changes on ECG, possible global or regional LV and/or RV dysfunction, and possible troponin T or I elevation;
- New onset or progressive HF, with impaired LV and/or RV systolic function and possibly non-specific ECG changes, AV or IV block, or ventricular arrhythmias;
- Life-threatening conditions including severe arrhythmias and aborted SCD, severe impairment of LV function, and cardiogenic shock (also known as fulminant myocarditis) requiring inotropic or mechanical circulatory support. Children and women may be more susceptible to present with fulminant myocarditis.

In a multicenter Italian registry of 443 patients with acute myocarditis, only 26.6% had complicated myocarditis at presentation, and this was associated with higher risk of cardiac death or heart transplantation in 5 years (30).

Some patients, on the other hand, may come to medical attention at a later stage of the disease, with chronic HF symptoms and signs that have developed over more than 3 months without a distinct onset.

Compared to those with acute myocarditis, patients with chronic myocarditis and chronic inflammatory cardiomyopathy are usually hemodynamically stable and present with only mild plasma troponin level elevation, often disproportionate to the severity of left ventricular dysfunction.

Common clinical tools are usually insufficient to diagnose myocarditis, so additional information from cardiac imaging techniques or EMB are necessary to confirm or exclude the disease.

Conducting EMB is often limited to severely ill patients with reduced left ventricular function because of its potential complications.

## Diagnosis

Transthoracic echocardiography (TTE) usually represents the first-line imaging method for suspected myocarditis, especially when hemodynamical instability precludes the use of more accurate imaging modalities such as CMR. TTE plays a fundamental role in excluding other causes of HF or chest pain, but it has limited diagnostic accuracy because of lack of specific echocardiographic findings. The most common findings at presentation are regional wall motion abnormalities (most commonly involving the inferior or inferolateral walls), diastolic dysfunction with preserved left ventricular ejection fraction (LVEF), and global left ventricular systolic dysfunction. The disease may also present with features resembling hypertrophic, dilated, or restrictive cardiomyopathies. A non-dilated, globally hypokinetic left ventricle with increased wall thickness and echogenicity (resulting from myocardial interstitial edema), possibly associated with right ventricular dysfunction, may be present in the case of fulminant myocarditis. In general, the presence of normal ventricular volumes rather than ventricular dilation can aid in distinguishing between acute myocarditis and chronic inflammatory cardiomyopathy (2, 28).

More recently, 2-dimensional speckle tracking echocardiography has emerged not only as a diagnostic tool but as a prognostic tool as well for patients with suspected acute myocarditis, even in the case of preserved LVEF at baseline and during follow-up (31).

In a study (2015) on 28 consecutive patients with CMR-verified diagnosis of acute myocarditis, global, epicardial, and endocardial longitudinal strains were found to be significantly correlated with the degree of myocardial edema detected by CMR. In the same study, a strain was found to be predominantly decreased in areas that showed greater extension of edema at CMR, namely, the infero-postero-lateral segments (32).

Reports have also shown that newer echocardiographic techniques such as real-time myocardial contrast echocardiography (RTMCE) can provide additional information in the setting of myocarditis by revealing attenuated perfusion with delayed contrast replenishment (presumably due to impaired microvascular integrity) in segments affected by the inflammatory process (33). However, the sensitivity and specificity of these new methods have yet to be adequately defined.

Nuclear imaging is not routinely recommended in the work-up of suspected myocarditis, owing to paucity of available data and overall low reported sensitivity. Nonetheless, scintigraphy with Indium-111 labeled antimyosin antibodies may be able to localize and visualize necrotic myocardial areas, in which loss of cellular membrane integrity leads to exposure of intracellular proteins.

18F-fluoro-2-deoxyglucose PET (18F-FDG-PET) is considered more sensitive for detection of metabolically active processes (including inflammation) and may be employed in selected cases, such as patients with contraindications to CMR or those with suspected cardiac sarcoidosis (28).

Multidetector computed tomography coronary angiography (MDCT) and the recently introduced delayed enhancement-MDCT(DE-MDCT) can be potentially employed to differentiate ischemic from non-ischemic cardiomyopathy in the same way as CMR.

CMR imaging has emerged as the non-invasive reference technique for diagnosis and follow-up of patients with myocarditis. The accuracy and reproducibility of cardiac structure evaluation, the unique ability of non-invasive tissue characterization, and the lack of ionizing radiation make CMR very attractive as a potential “all-in-one technique.” CMR provides valuable data to confirm or establish the diagnosis of myocarditis, screen subclinical cases, risk stratify patients according to established independent prognostic factors (e.g., LVEF, end-systolic-volume, and extent of myocardial edema), predict the prognosis, and monitor the response to therapy during follow-up (2, 28).

Diagnosis of myocarditis by CMR is based on the Lake Louise Criteria, which were first published in 2009 and recently updated in 2018.

The original criteria: myocardial edema, detected as increased signal intensity on T2- weighted images; hyperemia, corresponding to intense signal in early gadolinium enhancement images (EGE); necrosis or fibrosis, detected on late gadolinium enhancement (LGE) images. The presence of 2 out of the 3 criteria supported the diagnosis of myocarditis, with a sensitivity of 74% and a specificity of 86% (28).

Most acute myocarditis cases actually present with preserved LVEF and without regional wall motional abnormalities, thus tissue characterization by CMR is a crucial tool to aid in the diagnosis of the condition.

**FIGURE 2:**
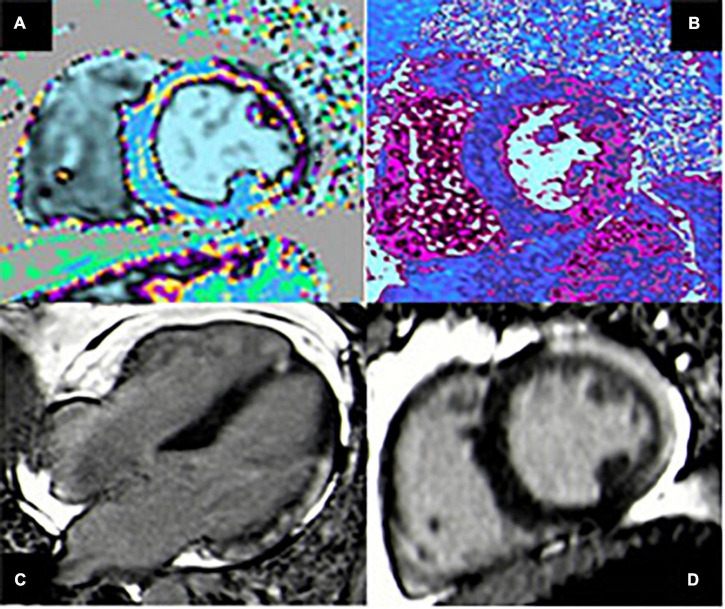
Case of acute myocarditis. (A,B) T1 and T2 mapping show increased value of both parameters in the lateral wall compatible with myocardial edema. (C,D) Four-chamber and short-axis views showing subepicardial late gadolinium enhancement (LGE) with typical pattern in the infero-lateral wall.

**FIGURE 3:**
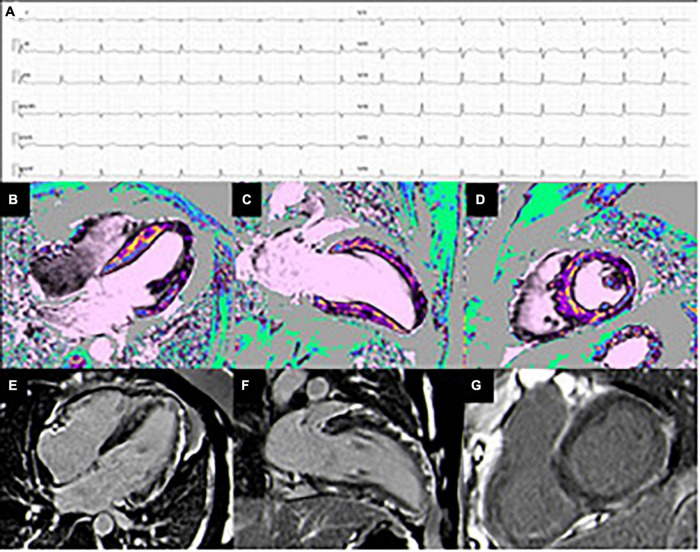
Case of a 50 y/o male with low-risk profile hospitalized for chest pain. (A) ECG pattern during chest pain showing PR-depression with diffuse ST-elevation. The coronary angiography was negative for significant lesions. (B–D) T1 mapping shows diffuse increased value more evident in the anterior and lateral wall, compatible with myocardial edema (4-chamber, 2-chamber, and short-axis views). (E–G) Extensive subepicardial LGE in the infero-lateral and in the antero-septal wall (4-chamber, 2-chamber, and short-axis views).

Distribution of LGE in myocarditis can be very heterogenous, but the most common patterns include patchy, non-contiguous lesions in the subepicardial layers of the left ventricular free wall, or intramural rim-like lesions in septal wall (34). These findings generally allow to exclude ischemic etiologies, in which LGE is typically found in the subendocardial layer (Figures 2, 3). According to a large Italian multicenter study, subepicardial LGE in the inferolateral wall was the most frequent LGE location regardless of the clinical pattern of myocarditis at presentation (HF, arrhythmias or infarct-like), while mid-layer left ventricular septal LGE was more common in patients with HF or arrhythmic presentation. Patients also presented with significantly higher number of segments with LGE, larger left ventricular volumes, lower LVEF, and lower RV systolic function than the infarct-like group (35).

**FIGURE 4:**
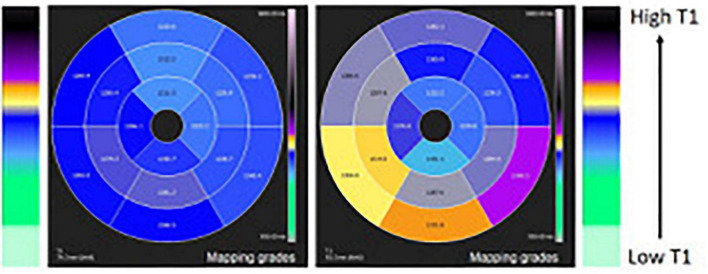
T1 mapping of the left ventricle 16 segment-model polar map comparing a normal case (left panel) and a case with higher T1 in the inferior wall due to inflammatory process (right panel).

The extension and distribution of LGE also have a relevant prognostic value, as discussed in detail in the last section of the manuscript. Over the last years, there have been important developments in the field of CMR tissue characterization owing to the advent of T1 and T2 mapping and extracellular volume quantification techniques. A T1 map is a parametric reconstructed image where the signal intensity of a single pixel represents the T1 longitudinal relaxation time of the corresponding myocardial voxel, according to its specific tissue characteristics (Figure 4). T1 mapping has several advantages over conventional CMR sequences, including higher sensitivity and ability to detect even early diffuse fibrosis not yet visible with LGE imaging, safety in the setting of renal insufficiency, heart rate independence, and lack of reliance on reference values. Disadvantages are mainly related to lack of standardization due to the emerging nature of the technique (36). In the same way, a T2 map is a CMR sequence used to calculate the T2 relaxation times of a certain tissue and display them on a parametric map (Figures 2, 3).

Considering the enhanced sensitivity of T1 and T2 mapping in detecting and quantifying myocardial fibrosis and edema, the Lake Louise Criteria were updated in 2018. Accordingly, when acute myocarditis is suspected, CMR findings are consistent with myocardial inflammation if both T1-based criteria (regional or global increase in myocardial T1, on native myocardial T1-mapping, extracellular volume quantification, or LGE imaging) and T2-based criteria (regional or global increase in myocardial T2 signal either on T2-weighted imaging or T2-mapping) are present. In an appropriate clinical scenario, the presence of only one between T1- or T2-based criteria may still support the diagnosis of acute myocarditis, even if with less specificity. Finally, the other criteria include pericardial effusion or high signal intensity of the pericardium in LGE images, T1 or T2-mapping, and wall motion abnormality (37).

These revised criteria have shown greater diagnostic performance, with improved sensitivity (87.5%) and specificity (96.2%) for diagnosis of acute myocarditis (28).

CMR mapping techniques may also improve the detection of tissue alterations in the case of subacute or chronic myocarditis when conventional T1- and T2-weighted images are often not sufficiently sensitive to detect subtle myocardial edema or fibrosis (36).

## Management of Myocarditis

Current recommendations for management of myocarditis mostly consist of non-specific therapies and are largely based on expert consensus, given the absence of large multicenter randomized controlled trials (2, 28, 29).

This manuscript focuses on viral-induced myocarditis, thus management of specific disease subtypes such as giant cell or eosinophilic myocarditis is beyond the scope of the study.

Hemodynamically stable patients with suspected myocarditis, even when asymptomatic or mildly symptomatic, should be initially admitted to a hospital for clinical monitoring given the existing but mostly unpredictable risk of evolution toward severe brady- or tachy-arrhythmias and decompensation. Some findings appear to be especially associated with increased risk of life-threatening arrhythmias, including persistent or fluctuating cardiac enzyme levels, sinus bradycardia, prolonged QRS duration, and progressive left ventricular motion abnormalities on echocardiography (2).

Especially for patients presenting with chest pain, elevated troponins and possibly ischemic ECG changes, invasive coronary angiography, or computed tomography angiography is often necessary to exclude an acute coronary syndrome (2, 28).

Myocarditis with HF-like presentation and ventricular dysfunction should be treated according to current HF guidelines. The appropriate timing for weaning from therapy after recovery of ventricular function, however, remains unclear (2, 38).

Beta-blockers are often employed in treatment of acute myocarditis, even in uncomplicated disease, presumably by virtue of the perceived protection they provide against arrhythmic events (28).

On the other hand, scientific societies recommend against the use of non-steroidal anti-inflammatory drugs, particularly acetylsalicylic acid, in the context of acute myocarditis. In fact, despite being the cardinal treatment for acute pericarditis, these drugs have been associated with increased mortality in experimental models of myocarditis, and data on their use in the clinical setting are scarce and inconclusive (2, 29).

It is widely agreed upon that physical activity should be limited during an acute disease, as it may increase the risk of sudden cardiac death. Accordingly, exercise testing is also contraindicated in the acute setting of myocarditis since it may precipitate arrhythmia. Athletes should refrain from taking part in competitive sports for at least 3 months after the onset of myocarditis regardless of age, sex, or severity of symptoms (39). Clinical re-evaluation, possibly with functional testing, is indicated before resuming participation in competitive sports (2, 29).

## Antiviral and Immunosuppressive Treatments

To date, no specific evidence-based treatment is available for virus-induced myocarditis. Since the pathogenesis of cardiac damage in myocarditis is often attributed to autoimmune/hyperimmune response to viral infection, immunomodulatory therapy has been considered potentially useful.

Preliminary data have shown that treatment with interferon-beta can promote viral clearance in patients with enteroviral or adenoviral myocarditis and may improve ventricular function and survival, but further studies are needed before this can be implemented in clinical practice (40, 41).

Similarly, treatment with acyclovir, ganciclovir, or valacyclovir may be considered in patients with fulminant herpes virus disease, although its efficacy has not yet been demonstrated in the context of myocarditis (2).

As mentioned, the causal role of Parvovirus B19 (B19V) in the pathogenesis of myocarditis is currently debated. However, three potential therapeutic strategies for B19V-related myocarditis have been proposed and are currently under investigation: high-dose intravenous immunoglobulin (IVIG), telbivudine, an antiviral nucleoside analog used primarily in retroviral and hepatitis B virus infections, and immunosuppressive therapy with prednisone and azathioprine (42).

IVIG is used in a number of autoimmune diseases and possesses anti-inflammatory as well as antiviral effects, thus representing a potential therapeutic strategy for both viral and autoimmune myocarditis.

The effects of IVIG were investigated in a prospective, double-blind, randomized, placebo-controlled study that enrolled 40 patients with chronic HF of various causes (i.e., ischemic and non-ischemic) who did not undergo EMB. In this study, IVIG use was associated with significant improvement in LV systolic function, as well as a significant increase in plasma levels of anti-inflammatory mediators (43).

Similarly, studies on the pediatric population have shown that the use of IVIG for treatment of acute myocarditis is associated with improved recovery of LV function and increased probability of survival during the first year after presentation (44).

Other investigators, however, found no improvement in LVEF with the use of IVIG in a cohort of patients with DCM, including 15% with biopsy-proven myocarditis (45).

Overall, the role of IVIG in viral myocarditis currently remains largely unknown.

Immunoadsorption is another potential therapy for treatment of myocarditis and has already been conducted on several antibody-mediated autoimmune diseases. So far, small randomized trials involving patients with idiopathic DCM have demonstrated improvement in LV systolic function and HF biomarker levels and reduction in myocardial inflammation by immunoadsorption (46).

Finally, immunosuppressive therapy is currently considered an option only for virus-negative, based on positive results of several studies (47). As mentioned, however, steroid and azathioprine therapy may also represent a possible future strategy for B19V-associated disease (48).

## Temporary Circulatory Support

Acute myocarditis remains a challenge for all clinicians because of its wide range of symptoms and unpredictable clinical course, which may result in decreased cardiac function and inability to maintain sufficient systemic pressures.

The initial therapy for patients in cardiogenic shock includes mechanical ventilatory support to reduce systemic oxygen consumption and support with inotropic or vasopressor drugs to improve contractility, pressure, and perfusion systems.

In cardiogenic shock, however, the use of high dosages of vasoactive drugs may lead to increased oxygen consumption without overall benefit on perfusion and outcome.

Thus, when medical therapy is unable to maintain adequate cardiac output, mechanical circulatory support is indicated.

In recent years, the use of temporary mechanical circulatory support, including intra-aortic balloon counterpulsation, veno-arterial extracorporeal oxygenation (ECMO), and more recently the Impella system, has been extended to treat patients with cardiogenic shock refractory to medical therapy, with the aim of ventricular chamber unloading, maintenance of coronary perfusion, and decongestion of venous circulation.

Because of its rapid setup, ECMO may be a suitable first choice for patients suffering from hemodynamic failure compared to the more complicated and time-consuming ventricular assisted device (VAD). Patients with acute myocarditis may also suffer from biventricular failure, and ECMO may be useful in this regard because of its ability to provide support to the right ventricle. In addition, ECMO may also serve as a screening tool to select the most suitable candidate for long-term VAD or heart transplantation (49).

Impella could be used in combination with ECMO to optimize left ventricular outflow and oxygenation and to allow early weaning from ECMO, as it can provide partial left ventricular support when ECMO is removed (50).

Early implantation of assistive devices in the setting of fulminant myocarditis has been suggested to improve outcomes not only through the primary function of mechanical circulatory support but also by reduction in the systemic inflammatory state (51). Overall, however, there is no evidence of superiority of one mechanical support system over the other or over medical therapy alone.

## Arrhythmias in Myocarditis

Among the extremely heterogeneous clinical manifestations of myocarditis, cardiac arrhythmias represent a major issue, determining a specific clinical entity defined as “arrhythmic myocarditis”, poorly described in medicalliterature (52). Indeed, a wide spectrum of bradyarrhythmias and tachyarrhythmias, harmless and/or potentially life-threatening, may characterize both the acute “hot” inflammatory stage and the chronic “cold” post inflammatory stage of myocarditis.

Data report that in up to 24% of cases, the first clinical manifestation of inflammatory heart disease consists of syncope, SCD, or arrhythmias not necessarily accompanied by cardiocirculatory decompensation or other signs of significant structural heart disease (53). In patients with acute myocarditis, non-sustained ventricular tachycardia is the most frequent event, with reported prevalence of 28%. Episodes of sustained ventricular tachycardia or ventricular fibrillation seem to fluctuate between 7.3 and 9.7% (54). Since either local or systemic inflammation has been associated with atrial fibrillation (AF) pathogenesis in the general population, it is not surprising that this arrhythmia represents a common manifestation of myocarditis, with reported prevalence of 2.5–14% (55). Other supraventricular tachycardia and atrioventricular blocks (AVB) are less frequent, with their prevalence ranging from.8 to 1.7–10%, respectively (56, 57). Regarding bradyarrhythmias, the female gender has been found to be independently associated with the occurrence of AVB and advanced AVB in patients with myocarditis, while only high-degree AVB resulted to be independently associated with increased morbidity and mortality in this clinical scenario (57).

Historically, myocarditis has been considered responsible for a large proportion of SCD, especially in male patients younger than 40 years old without prior recognized structural heart disease (54). Despite the true occurrence being poorly characterized, the prevalence of undiagnosed myocarditis in post-mortem series ranges from 9 to 44%, involving 2% of infants, 5% of children, and 4–8% of athletes (58, 59).

The etiology of myocarditis determines the risk of arrhythmic events, especially in the acute stage of the disease and more frequently in non-lymphocytic myocarditis. Indeed, both ventricular/supraventricular tachyarrhythmias and bradyarrhythmias are more commonly associated with infrequent non-viral myocarditis, such as giant cell myocarditis (GCM) and cardiac sarcoidosis (CS)-related myocarditis, which express prevalence of ventricular arrhythmias of 29 (60) and 55% (61), respectively. In particular, GCM should be suspected when episodes of arrhythmic storms are refractory to antiarrhythmic drug therapy. Instead, in the presence of simultaneous pericardial inflammatory involvement (myopericarditis or perimyocarditis), arrhythmias are significantly less common (overall prevalence < 10%) and, more frequently, of supraventricular origin (62).

Viral myocarditis with lymphocytic infiltrate accounts for a large proportion of arrhythmic myocarditis and SCD (63). Of note, among viral myocarditis, HIV-related myocarditis is more frequently associated with documented arrhythmias (64) than more common infections such as enterovirus (including *Coxsackie B virus)*, adenovirus, parvovirus B19, and human herpes virus 6. In this clinical context, athletes with acute viral myocarditis represent a population with particularly higher risk of experiencing ventricular arrhythmias and SCD (59). While routine physical activity is considered to improve immunological defenses, strenuous and prolonged training, typical of professional athletes, seems to lower the antiviral immunity, probably reducing salivary secretory immunoglobulin A, lactoferrin, and lysozyme, and altering T cell response (55). Furthermore, some studies conducted on murine models of viral myocarditis (e.g., coxsackievirus B3) have shown that intense sport activity may enhance pathogen agents virulence, increasing both the extent of myocardial cell necrosis and overall mortality (65). Thus, it is not surprising that despite the implementation of systematic pre-participation cardiological screening, the incidence of SCD due to acute or fulminant myocarditis in the athlete is around 10% of all fatalities (66).

## Potential Mechanisms of Arrhythmia in Myocarditis

Currently, the exact mechanisms responsible for arrhythmogenesis in myocarditis are unclear and still a matter of debate. Several molecular and immunopathogenic mechanisms are likely involved in the disease process both in the acute “hot” phase and in the chronic “cold” phase (Table 2). Indeed, the main postulated hypotheses to explain the arrhythmogenicity of the acute phase of viral myocarditis are: direct pathogen-mediated cytolysis determining electrical instability (52); myocardial edema: local inflammation, cytokine release, and cell death constitute electrically sensitive regions to trigger activity and abnormal automaticity (67); alteration in myocardial expression of connexin proteins with consequent gap junction dysfunction and dysregulation (68); acute ischemia triggered by viruses with endothelial tropism (e.g., Parvovirus B19) leading to coronary macrovascular or microvascular disease or prolonged vasospasm (52); induced abnormal calcium handling (69); viral-related ion channel impairment with decreased Kv4.2 potassium channel expression (70), which can partially explain the reported association between acute myocarditis and ventricular arrhythmia in myocardial channelopathies, such as Brugada syndrome, short QT syndrome, and early repolarization syndrome; an inflammatory component is often identified in the myocardium of individuals with post-mortem diagnosis of arrhythmogenic cardiomyopathy (AC) (71). Conversely, desmosomal genetic mutations, responsible for AC, can predispose to ventricular arrhythmias in myocarditis (72). However, different data suggest that myocarditis may frequently lead to structural changes that can mimic AC. For this reason, some authors suggest that clinicians should consider genetic testing in patients presenting with acute arrhythmic lymphocytic myocarditis (73).

**TABLE 2:** Different molecular and immunopathogenic mechanisms involved in the disease process according to the disease phase: acute “hot” versus chronic “cold”.

Mechanisms of arrhythmia in the acute “hot” phase	Mechanisms of arrhythmia in the chronic “cold” phase
– Direct pathogen-mediated cytolysis	– Persistent active chronic inflammation
– Myocardial oedema, cytokines release, and cell death	
– Gap junction dysfunction due to altered connexins expression (typical of Coxsackievirus B3)	– Post inflammatory myocardial scar formation
– Acute ischemia, microvascular disease and prolonged vasospasm (typical Parvovirus B19)	
– Abnormal calcium handling	– Residual ventricular dysfunction
– Ion channel impairment (typical of myocardial channelopathies)	
– Unmasking of structural genetic cardiomyopathy (e.g., AC)	– Electrical remodeling

*AC, arrhythmogenic cardiomyopathy.*

Instead, arrhythmias in the late stage of inflammatory cardiomyopathy rarely result from persistent active inflammation but rather from post-inflammatory myocardial scar formation, residual ventricular dysfunction, and electrical remodeling. The healing process of active acute myocarditis can lead to fibrosis typically involving subepicardial/mid myocardial layers of the infero-lateral left ventricular wall with a peculiar “band” pattern. This late viral-induced myocardial fibrosis determines the formation of re-entry circuits, regional slowing of action potentials, and suitable substrates for life-threatening ventricular tachyarrhythmias even in subjects with normal LVEF (74).

The difference in the arrhythmogenic mechanism between the acute and late phases is corroborated by different anatomopathological findings, which may induce different arrhythmic manifestations. Indeed, myocardial necrosis with massive inflammatory infiltrates are common in acute myocarditis, whereas replacement fibrosis, still accompanied by leukocyte infiltration with no myocyte necrosis, is typical of the chronic “cold” phase of myocarditis (28). As demonstrated by Peretto et al., these anatomopathological and arrhythmogenic differences clinically manifest themselves with two different arrhythmic patterns: polymorphic and irregular ventricular arrhythmias are more common during the active inflammatory phase, whereas monomorphic and regular ventricular arrhythmias, suggesting a static and “cold” substrate, are associated with healed myocarditis. Similarly, a French study found that ventricular fibrillation was the most common initial ventricular arrhythmia in acute myocarditis setting (58%), that ventricular tachycardia was the most common in myocarditis sequelae (78%), and that cardiorespiratory arrest was twice as frequent in the course of acute myocarditis (68 versus 30%) (75).

## Short-Term Prognosis and Treatment of Arrhythmic Myocarditis

In acute myocarditis, regardless of etiology, asymptomatic non-sustained ventricular tachycardia, premature ventricular beats, or premature atrial beats are generally considered benign and should not be treated with any anti-arrhythmic medication accordingly, whereas symptomatic non-sustained VTs can be treated with beta-blockers and antiarrhythmic medications such as amiodarone and mexiletine (76).

In contrast, patients with acute myocarditis with refractory life-threatening ventricular arrhythmias show an adverse short-term prognosis. Indeed, patients with major arrhythmias in the context of acute myocarditis have been proven to more likely (odds ratio = 7.59) require mechanical assist device use, heart transplantation and to experience a SCD (77). In the pediatric population, the finding of significant tachyarrhythmias was associated with 2.3 times increase in odds of mortality, 58% increase in length of hospitalization, and 28% increase in costs per day (78). Therefore, in fulminant myocarditis, the short-term prognosis is largely dependent on an early treatment and on the appropriateness in the use of life-saving devices, which include in some cases the transfer to Hub experienced in circulation mechanical support (58). Moreover, in this clinical scenario, i.e., presence of arrhythmic events associated with hemodynamic instability, it is mandatory, according to a 2020 Expert Consensus document, to perform an EMB, an invasive but low risk (1–2% for cardiac complications) procedure considered the reference standard for diagnosis of myocarditis, since it allows to identify the proper underlying mechanisms and decide for appropriate therapy (28). The execution of EMB allows to reach a definite diagnosis and thereby adopt a targeted therapy (if available) based on the etiology detected. Moreover, EMB allows to distinguish between the presence or absence of inflammatory involvement, acute or chronic phase of myocarditis, and virus-negative or virus-positive inflammatory cardiomyopathy. Furthermore, among the types of virus-positive inflammatory cardiomyopathy, EMB allows to differentiate between virus-induced active myocarditis (i.e., caused by adenoviruses or enteroviruses) and virus- associated myocarditis (whether the virus is a bystander is not clear; i.e., in the case of latent infections with herpesviruses or B19V) (3). For example, in the case of B19V which can also be found in the heart of healthy patients, EMB allows for the analysis of DNA copy number and VP1/VP2 RNA expression (representing transcriptional activity) to define whether the myocarditis is definitely caused or not by the virus itself (79).

Other recommended clinical indications to perform EMB in the setting of acute or chronic myocarditis are the following: myocarditis presenting with or complicated by severe HF, cardiogenic shock, or high-degree AVB; suspected chronic inflammatory cardiomyopathy, especially if associated with peripheral eosinophilia; myocarditis or chronic inflammatory cardiomyopathy with persistent or relapsing release of biomarkers of myocardial necrosis; myocarditis in the setting of immune checkpoint inhibitor therapy, where appropriate diagnosis has implications for patients receiving additional cancer therapy.

To enhance the diagnostic yield of EMB in the setting of myocarditis, it is recommended to perform it within 2 weeks of symptom onset and to collect from 4 to 6 specimens (28).

Tachyarrhythmias are not the only arrhythmias that lead to poor outcomes in viral myocarditis. As already mentioned, the development of high degree AVB during acute myocarditis has been found to be independently associated with increased incidence of cardiogenic shock, respiratory failure, renal failure, and mortality in a registry of 31,760 patients, while non-advanced AVB appeared as a benign clinical entity (57). During the acute phase, conduction abnormalities such as complete AVB or symptomatic bradycardia can often be transient, making temporary pacemaker the perfect first step treatment (74). On the other hand, guidelines suggest that a permanent pacing system should be implanted if complete AVB or symptomatic bradycardia does not resolve after several days (generally 5–7 days) of monitoring and the patient is otherwise ready to be discharged home (67, 80).

A fundamental complementary therapeutic-prophylactic step in short-term management of acute arrhythmic myocarditis is avoidance of even mild exercise for a time period that has been shortened from 6 to 3 months in recent recommendations but can be extended to 6 months according to the clinical severity and duration of illness, left ventricular function, and extent of the inflammatory process on CMR (39).

## Long-Term Prognosis and Implantable Defibrillator Indications in Arrhythmic Myocarditis

When promptly managed, acute-phase arrhythmias tend to be self-limiting and do not bear a significant long-term prognostic value, so European guidelines suggest waiting for the resolution of the acute phase before evaluating the appropriateness of implantable cardioverter defibrillator therapy in secondary prevention (2, 81). It is generally assumed that far from the acute phase of myocarditis, the supposed transient inflammatory pro-arrhythmogenic trigger disappears, similarly to the ischemia pro-arrhythmogenic trigger in the acute phase of a myocardial infarction. This is because myocarditis has long been considered a fully reversible disease. Actually, recent findings from different groups strongly suggest that arrhythmic events in acute myocarditis are associated with long-term poor outcomes, including significant arrhythmia recurrence and SCD, supporting the concept that early ICD implantation in patients presenting sustained ventricular arrhythmias in the acute phase of myocarditis could be extremely helpful. Indeed, data from the Multicenter Lombardy Registry showed that patients with complex ventricular arrhythmias at presentation were at higher risk of worse long-term outcomes than uncomplicated myocarditis cases that had, instead, benign long-term prognosis and low risk of subsequent arrhythmic events and left ventricular systolic dysfunction (30). Similarly, a French study (75) showed that patients presenting with ventricular tachycardia or ventricular fibrillation in the acute phase of myocarditis who had received ICD implantation early for secondary prevention had a very high risk (39%) of recurrence of major arrhythmic ventricular events (MAEs) over a median follow-up period of 3 years. In addition, an alarming 80% of patients in a study subgroup who preferred not to have ICD implantation experienced an MAE over time. These results show that the risk of MAE recurrence remains high after resolution of the acute episode, questioning the notion that myocarditis is a fully reversible disease. Furthermore, it is interesting to note that the first MAE occurred after the first 3 months of the index event in 82% of patients; these data disagree with the theory that the risk of arrhythmia is reduced once the acute phase of myocarditis is resolved and challenges the utility of wearing a wearable cardiac defibrillator (WCD) for 3 months after the initial arrhythmic event as recommended by some teams (82). In accordance with these conclusions, the results of an Italian population in which 54% of patients who received an ICD in secondary prevention for an MAE that occurred during acute myocarditis had major ventricular arrhythmias requiring ICD intervention during an average follow-up of 65 months (83).

Considering this new evidence, despite the paucity of long-term longitudinal data on mortality and morbidity, we suggest that it is reasonable to propose to patients who present with sustained VT or VF the implantation of ICD in secondary prevention prior to discharge for the acute episode, without waiting for the resolution of the supposed transient inflammatory pro-arrhythmogenic trigger even with the use of a WCD. As already mentioned, life-threatening arrhythmic myocarditis is an unfortunate eventuality among professional athletes, as in the case of footballer Christian Eriksen who gained great resonance in the media during the 2020 European Football Cup. In athletes, ICD programming should include high-rate cut-offs and long-detection duration in order to reduce inappropriate shocks during physical exercise (84).

Thanks to most recent technological progresses, in all cases when pacing is not needed because of coexisting bradyarrhythmias, a subcutaneous ICD (S-ICD) should always be considered as a reliable alternative to a transvenous (TV)-ICD because of easier management of lead and generator-related complications (85). Considering S-ICD over TV-ICD is of the utmost importance in this clinical scenario, since myocarditis often affects young patients (especially when compared to other cardiac diseases leading to ICD implantation) who may pay the highest price of TV-lead related complications, potentially facing several years of device therapy. Indeed, S-ICD has been recently proven safe and effective in teenagers/young adults, showing similar rates of inappropriate shocks and complications when compared to the older population (86).

On the other hand, primary prevention patients, i.e., patients with acute myocarditis who acutely developed impaired LVEF (LVEF ≤ 35%), without MAE occurrence, had different indications. In this clinical scenario, doubts regarding the need for early ICD placement are more consistent, at least in the critical period of therapeutic optimization for ventricular dysfunction. A recent German study (87) proposed the use of WCD following good results of WCD therapy in patients with LVEF ≤ 35% post-myocardial infarction or in patients with newly diagnosed cardiomyopathy still undergoing optimal medical therapy within 90 days after diagnosis (88). The authors found that MAE requiring WCD intervention occurred in approximately 20% of patients with an estimated necessity for WCD wearing until MAE occurrence of 86.4 days. It should be highlighted that this study also included a group of patients with WCD for an acute episode of DCM and impaired LVEF in the absence of myocardial inflammatory involvement; in this population, only 3% of the patients experienced an MAE requiring WCD intervention with a calculated necessary WCD wearing time until MAE occurrence of 6.5 years (87). These data emphasize how myocardial inflammation exposes patients to a greater arrhythmic burden than other etiology and suggests a possible role of WCD as a bridge to LVEF in patients with acute myocarditis and impaired LVEF without MAE. Instead, a similar study (89)found a significantly lower rate of MAE, about 3% over an average wearing time of 86 days. However, it should be noted that only one-third of patients had recovered LVEF > 35% at the end of the WCD wearing time and, therefore, had no indication for subsequent prophylactic ICD placement. These data pave the way for earlier indication of ICD implantation and highlights the need to identify reliable predictors of LVEF recovery and MAE occurrence.

It is essential to discuss ICD implantation indications in the setting of chronic inflammatory cardiomyopathy as well, both in terms of primary and secondary preventions. With regard to primary prevention, i.e., patients with chronic inflammatory cardiomyopathy and impaired LVEF (LVEF ≤ 35%) without MAE occurrence, indications for prophylaxis with ICD therapy are those suggested by the guidelines for patients affected by HF with reduced ejection fraction (38). It should be emphasized that in the literature, about 30% of patients with chronic inflammatory cardiomyopathy required an ICD or cardiac resynchronization therapy-defibrillator device, and that half of them experienced at least one episode of ventricular arrhythmias with appropriate ICD therapy over time (90). Therefore, even in the context of chronic and non-acute inflammation, it emerges that myocardial inflammation exposes patients to a significantly high arrhythmic burden: 21% of patients with chronic myocardial inflammation had at least one MAE within the first year after ICD implantation compared with the no events reported in patients with DCM without inflammatory involvement (90).

Finally, as for secondary prevention, regardless of presence or absence of ventricular dysfunction, the International Guidelines agree on the mandatory need for ICD in cases of sustained VT or VF (76, 81). Recent data further stress the need for ICD implantation in patients with chronic inflammatory cardiomyopathy for secondary prevention: over a median follow-up period of 3 years, MAE occurred in 60% of patients with chronic inflammatory cardiomyopathy with a Kaplan–Meier MAE rate estimates at 1 and 3 years of follow-up of 43 and 64%, respectively.

Table 3 summarizes primary and secondary prevention ICD indications suggested by our group and/or by International Guidelines according to the acute or chronic clinical scenario of myocarditis presentation.

**TABLE 3:** Primary and secondary ICD prevention indications according to the clinical scenario of presentation.

Primary prevention	Acute phase of myocarditis	Chronic phase of myocarditis
	– Consider WCD as a bridge during therapy optimization (∞) – Consider earlier ICD implantation (∞)	– ICD implantation according to International HF Guidelines (*)
**Secondary Prevention**	**MAE in the acute phase of myocarditis**	**MAE in the chronic phase of myocarditis**
	– ICD implantation suggested prior to discharge (∞) – WCD for three months not suggested (∞)	– ICD implantation mandatory by International Guidelines (*)

ICD, implantable cardiac defibrillator; WCD, wearable cardiac defibrillator; HF, heart failure, MAE, major arrhythmic ventricular events. *, corroborated by International Guideline indications; **∞**, suggest by our group of work, deserve to be addressed in future clinical research.

## Predictors of Arrhythmias in Myocarditis From Cardiac Imaging

The role of cardiac imaging in predicting the risk of arrhythmia in myocarditis is well-defined for echocardiography, as for the evaluation of LVEF, but considering that systolic function is usually preserved, other parameters have gained interest in the last years.

LGE is a powerful predictor of adverse events besides playing a fundamental role in diagnosis. LGE at CMR imaging within 5 days after initial presentation was strongly associated with adverse outcomes (all-cause death, cardiac death, or SCD) in 222 patients with biopsy-proven myocarditis over a median follow-up of 4.7 years; interestingly, patients without LGE did not experience SCD even if LVEF was severely impaired (91). In another study, LGE was independently associated with the composite adverse outcome of death, HF hospitalization, transplantation, sustained ventricular tachycardia, and recurrent myocarditis in 670 patients with suspected myocarditis over a median follow-up of 4.7 years: in particular, septal and midwall LGE showed the strongest association with MACE, patchy distribution portended to a near 3-fold increased hazard to MACE, and LGE extent (per 10% increase) corresponded to a 79% increase in risk of MACE (92).

Similar observations were derived from a multicenter Italian study, the ITAMY (ITAlian study in MYocarditis) registry, on 374 patients with myocarditis and preserved ejection fraction: over a median follow-up of 4.3 years, the presence of LGE (particularly in the anteroseptal mid-wall) was independently associated with a composite endpoint of HF hospitalization, sudden death, or implantable cardioverter-defibrillator shock (93). In a subgroup of 187 patients from this registry, however, the importance of performing a follow-up CMR after 6 months from the onset of symptoms to better stratify the prognosis of patients with myocarditis has been highlighted. Indeed, the presence of LGE in the acute setting is not necessarily synonymous to an irreversible damage, since it often reflects the edema of the first phase, which actually completely resolves at follow-up in the majority of cases. Overall, it was found that the presence of LGE (especially if in the mid-wall septum) without edema at 6-month CMR portended a worse prognosis, possibly by representing definite fibrosis. On the other hand, the concomitant presence of LGE and edema suggested active inflammation and chance of future recovery (94).

A recent metanalysis confirmed that in acute myocarditis, LGE presence at baseline CMR is independently associated with MACE (all-cause mortality, cardiac mortality, and major adverse cardiovascular events, including sustained ventricular arrhythmias) with a 3-fold increase in risk during a mean 2-year follow-up; the risk of experiencing MACE was doubled in patients with more extensive LGE (> 2 LV segments with LGE or LGE > 10% of LV mass or LGE > 17 g) and in patients with anteroseptal location of LGE (95).

Another interesting point is the measurement of abnormal myocardial mechanics, particularly left ventricular global longitudinal strain (GLS), assessed with both speckle tracking echocardiography and feature tracking techniques at CMR.

GLS in myocarditis resulted to be predictive of non-sustained ventricular tachycardias (cut-off value ≥ 12%) and was significantly lower in patients with myocarditis and cardiovascular events (arrhythmias, HF, cardiogenic shock, and syncope) during hospitalization (96).

Regarding CMR, a relationship between feature tracking strain parameters and adverse cardiac events (cardiac death, transplantation, implantable cardioverter-defibrillator or insertion of pacemaker, hospitalization, or stroke) over a follow-up of 41 months has been demonstrated in acute myocarditis (97); also, in a large cohort of patients with acute or subacute myocarditis feature tracking–derived GLS was associated with adverse cardiac events (hospitalization for HF, sustained ventricular tachycardia, or death) at a median follow-up of 3.9 years, incremental to some common clinical and CMR variables including ejection fraction and LGE extent (98).

Besides the role of LGE presence and abnormal GLS in variably defining a worse prognosis, an important point is that the absence of LGE and/or abnormal GLS should suggest a favorable prognosis in patients with myocarditis.

Newer techniques such as T1, extracellular volume, and T2 mapping, and their association with arrhythmic events, need to be better defined.

## Author Contributions

FS and SC contributed to conception and design of the study. EG, AM, AF, and EG wrote the first draft of the manuscript. MS and LI wrote sections of the manuscript. All authors contributed to manuscript revision, read, and approved the submitted version.

## Conflict of Interest

The authors declare that the research was conducted in the absence of any commercial or financial relationships that could be construed as a potential conflict of interest.

## Publisher’s Note

All claims expressed in this article are solely those of the authors and do not necessarily represent those of their affiliated organizations, or those of the publisher, the editors and the reviewers. Any product that may be evaluated in this article, or claim that may be made by its manufacturer, is not guaranteed or endorsed by the publisher.
